# Genetic Spectrum and Clinical Heterogeneity of Chinese Frontotemporal Dementia Patients: Data from PUMCH Dementia Cohort

**DOI:** 10.3233/JAD-220594

**Published:** 2022-09-27

**Authors:** Liling Dong, Jie Wang, Caiyan Liu, Jie Li, Chenhui Mao, Xinying Huang, Shanshan Chu, Bin Peng, Liying Cui, Jing Gao

**Affiliations:** Neurology Department, State Key Laboratory of Complex Severe and Rare Diseases, Peking Union Medical College Hospital, Chinese Academy of Medical Sciences and Peking Union Medical College, Beijing, China

**Keywords:** Behavioral variant of frontotemporal dementia, frontotemporal dementia, primary progressive aphasia, *TBK1*

## Abstract

**Background:**

There are relatively few data on the genetic spectrum of Chinese frontotemporal dementia (FTD) population.

**Objective:**

With the dementia cohort of Peking Union Medical College Hospital, we aim to illustrate the genetic spectrum of FTD patients, as well as the phenotypic heterogeneity of FTD-gene variant carriers.

**Methods:**

204 unrelated, clinically diagnosed FTD patients of Chinese ancestry were enrolled. All the participants received demographic survey, history inquiry, physical examination, cognitive assessment, blood biochemical test, brain CT/MRI, and gene sequencing.

**Results:**

56.4% (115/204) participants were clinically diagnosed with behavioral variant of FTD, 20.6% (42/204) with nonfluent/agrammatic variant primary progressive aphasia (PPA), 20.1% (41/204) with semantic variant PPA, and 2.9% (6/204) with mixed variant PPA. 11.8% (24/204) subjects harbored the potential causative variants in FTD-related genes, including the *MAPT* (*n* = 7), *TBK1* (*n* = 7), *GRN* (*n* = 2), *TBK1+GRN* (*n* = 1), *VCP* (*n* = 1), *TARDBP* (*n* = 1), *UBQLN2* (*n* = 1), *SQSTM1* (*n* = 1), *DCTN1* (*n* = 1), *HNRNPA1* (*n* = 1), and *C9orf72* GGGGCC repeats (*n* = 1). The *TBK1* T31fs, T457fs, K622fs, c.359-1G>A, the *VCP* P188T, and the *GRN* P50fs, P439fs were novel pathogenic/likely pathogenic variants. The *TBK1* carriers showed a later disease onset and a higher incidence of parietal atrophy relative to the *MAPT*carriers.

**Conclusion:**

There is genetic and clinical heterogeneity among Chinese FTD population. The *TBK1* has a high mutation frequency in Chinese FTD patients.

## INTRODUCTION

Frontotemporal dementia (FTD) represents a group of neurodegenerative diseases related to frontotemporal lobar degeneration. It is heterogeneous in phenotype, pathology, and genotype. The common phenotypes are behavioral variant of FTD (bvFTD) and primary progressive aphasia (PPA). The latter mainly comprises nonfluent/agrammatic variant PPA (nvPPA) and semantic variant PPA (svPPA). The common molecular bases include tau, TDP-43, and FUS proteins.

The *MAPT*, *GRN*, and *C9orf72* are the main causative genes of FTD. However, the genetic variants in the *MAPT*, *GRN*, and *C9orf72* can only account for about half of the familial FTD patients [1]. The previous studies reveal that some other genes are linked to FTD, including the *VCP*, *CHCHD10*, *TBK1*, *CHMP2B*, *TARDBP*, *SQSTM1*, *FUS*, *UBQLN2*, *OPTN*, *TREM2*, *CYLD*, *PRKAR1B*, *TIA1*, *TUBA4A*, *CCNF*, *DCTN1*, *HNRNPA2B1*, and *HNRNPA1* [1, 2].

There are relatively few data on the genetic spectrum of Chinese FTD population. In 2021, Dr. Shen reviewed the 97 genetic studies of Chinese FTD population. 32 rare variants in the *MAPT*, *GRN*, *C9orf72*, *CHCHD10*, *VCP*, and *TBK1* were found [3]. These mutations are definitely not sufficient to cover the genetic spectrum of Chinese FTD patients.

This is a retrospective study from the dementia cohort of Peking Union Medical College Hospital (PUMCH). Herein, we aim to expand the genetic reservoir of Chinese FTD population by interpreting the genetic spectrum in the clinically diagnosed FTD patients. In addition to the common *MAPT*, *GRN*, *C9orf72*, *CHCHD10*, *VCP*, and *TBK1* genes, we also focus on some rare FTD-related genes, including the *CHMP2B*, *TARDBP*, *SQSTM1*, *FUS*, *UBQLN2*, *OPTN*, *TREM2*, *CYLD*, *PRKAR1B*, *TIA1*, *TUBA4A*, *CCNF*, *DCTN1*, *HNRNPA2B1*, and *HNRNPA1*. Furthermore, we aim to illustrate the phenotypic characteristics of the FTD-gene mutation carriers. These will help us to better understand the phenotypic and genotypic heterogeneity of FTD patients.

## METHODS

### Participant enrollment

204 unrelated participants of Chinese ancestry were enrolled from the PUMCH dementia cohort between 2007 and 2021. The inclusion criteria were as the following: 1) Intact data on demographic survey, history inquiry, physical examination, cognitive assessment, blood biochemical test, brain CT/MRI, and gene sequencing; 2) Met the clinical diagnostic criteria for probable bvFTD, nvPPA, svPPA, and mixed variant PPA (mvPPA) [4–7]; 3) Informed consent for participation in this research wasobtained.

This study was approved by ethics committee of PUMCH (No. JS-1836).

### Gene sequencing and interpretation

The genomic DNA was extracted from the peripheral blood. The DNA libraries were sequenced on the Illumina platform (Illumina Inc., San Diego, CA, USA). The clean reads were aligned to the reference human genome (GRCh37/hg19) with the Burrows-Wheeler Aligner [8]. The annotation was performed with ANNOVAR (2017June8) [9]. The GGGGCC repeat expansion in the *C9orf72* was determined by the triplet repeat primed PCR with capillary electrophoresis.

This report focused on the GGGGCC repeat expansion in the *C9orf72*, as well as the rare variants in the *MAPT*, *GRN*, *CHMP2B*, *VCP*, *TARDBP*, *SQSTM1*, *FUS*, *UBQLN2*, *OPTN*, *CHCHD10*, *TBK1*, *TREM2*, *CYLD*, *PRKAR1B*, *TIA1*, *TUBA4A*, *CCNF*, *DCTN1*, *HNRNPA2B1*, and *HNRNPA1*. The rare variants met the following: 1) The minor allele frequency <0.0001 in the 1000genome, ESP6500, and GnomAD databases; 2) Nonsynonymous mutations located in the coding region or±1/2 splice site; 3) Interpreted as pathogenic, likely pathogenic, or uncertain significance according to the standards of American College of Medical Genetics and Genomics (ACMG) [10].

### Statistical analysis

The categorical variables were compared with the Chi-square or Fisher’s exact test. The continuous variables were assessed with the Analysis of Variance.

## RESULTS

### Cohort introduction

204 unrelated participants were enrolled. They were randomly recruited from different pedigrees all over China. 52.0% (106/204) were males and 48.0% (98/204) were females. 73.5% (150/204) subjects were early-onset (age of onset (AOO) <65 years old), while 26.5% (54/204) were late-onset (AOO ≥65 years old). 39.7% (81/204) subjects had a positive family history of dementia. That is, at least one of their first-degree or second-degree family members had dementia.

56.4% (115/204) subjects were clinically diagnosed with bvFTD, 20.6% (42/204) with nvPPA, and 20.1% (41/204) with svPPA. 2.9% (6/204) subjects showed both agrammatism and word comprehension impairment within two years of disease onset. They were diagnosed with mvPPA. Motor neuron disease (MND) coexisted in 2.5% (5/204) subjects, including three bvFTD, one nvPPA and one svPPA patient. None of the subjects developed schizophrenia, corticobasal syndrome or progressive supranuclear palsy.

The demographic data was shown in Table 1. The svPPA patients showed a higher frequency of sporadic cases than bvFTD, nvPPA, and mvPPA patients (80.5% versus 52.2%, 61.9%, 66.7%, *p* = 0.012). The gender, AOO, disease course, and APOE-*ɛ*4 status did not differ among the four subgroups.

**Table 1:** Demographics of the FTD participants

	FTD (*n* = 204)	bvFTD (*n* = 115)	nvPPA (*n* = 42)	svPPA (*n* = 41)	mvPPA (*n* = 6)	*p* ^*^
Male/Female	106 (52.0)/98 (48.0)	59 (51.3)/56 (48.7)	20 (47.6)/22 (52.4)	24 (58.5)/17 (41.5)	3 (50.0)/3 (50.0)	0.793
Age (y)	62.5±10.6	63.2±11.3	62.5±10.2	60.6±9.4	61.3±6.4	0.584
AOO (y)	59.1±10.1	59.7±10.7	59.2±9.9	57.9±9.3	57.3±5.9	0.778
Disease course (y)	3.3±2.6	3.6±2.7	3.3±3.1	2.7±1.4	4.0±2.0	0.490
Early/late-onset *n* (%)	150 (73.5)/54 (26.5)	80 (69.6)/35 (30.4)	31 (73.8)/11 (26.2)	34 (82.9)/7 (17.1)	5 (83.3)/1 (16.7)	0.400
FHD – /+ *n*(%)	123 (60.3)/81 (39.7)	60 (52.2)/55 (47.8)	26 (61.9)/16 (38.1)	33 (80.5)/8 (19.5)	4 (66.7)/2 (33.3)	0.012
*APOE* *ɛ*4 – /+ *n*(%)	156 (76.5)/48 (23.5)	89 (77.4)/26 (22.6)	30 (71.4)/12 (28.6)	31 (75.6)/10 (24.4)	6 (100.0)/0 (0.0)	0.557
PCV +/– (%)	24 (11.8)/180 (88.2)	7 (6.1)/108 (93.9)	8 (19.0) /34 (81.0)	9 (22.0)/32 (78.0)	0 (0.0)/6 (100.0)	0.016

FTD, frontotemporal dementia; bvFTD, behavioral variant of FTD; nvPPA, nonfluent/agrammatic variant primary progressive aphasia; svPPA, semantic variant primary progressive aphasia; mvPPA, mixed variant primary progressive aphasia; AOO, age of onset; FHD, family history of dementia; *APOE*, apolipoprotein E; PCV, potential causative variant; ^*^The variables were compared among the bvFTD, nvPPA, svPPA, and mvPPA patients.

### Mutation interpretation

20 rare variants in nine FTD-related genes were found (Table 2). According to the ACMG criteria, there were 11 pathogenic or likely pathogenic variants (PLPV) and nine variants of uncertain significance (VUS). Among the 11 PLPV, the *MAPT* L583V, P618L, the *TBK1* T462fs and the TARDBP I383V were reported before [11–13]. Seven PLPV were novel, including the TBK1 T31fs, T457fs, K622fs, c.359-1G>A, the *VCP* P188T, and the *GRN* P50fs, P439fs. Among the nine VUS, the *MAPT* Q561P, *SQSTM1* E362K, and *UBQLN2* P500S were reported before [11, 14, 15]. Six VUS were novel, including the *TBK1* T331N, M719V, R271W, the *GRN* T220I, the *HNRNPA1* N50S and the *DCTN1* R292H. No rare variants were found in the *CHCHD10*, *CHMP2B*, *FUS*, *OPTN*, *TREM2*, *CYLD*, *PRKAR1B*, *TIA1*, *TUBA4A*, *CCNF*, and *HNRNPA2B1*.

**Table 2:** Mutation interpretation

Gene	Variant	1000genome/ESP6500/GnomAD	SIFT/Polyphen/Mutationtaster	Interpretation	Clinvar	ACMG	Publication (PMID)
*MAPT*	L583V	-/-/-	D/D/A	PHA03247 domain	P	LP	12509859
	P618L	-/-/-	D/D/D	PHA03247 domain	P	P	9641683
	Q561P	-/-/-	T/D/D	PHA03247 super family domain	–	VUS	33006106
*TBK1*	T457fs	-/-/-		TBK1_CCD1 domain	–	LP	–
	K622fs	-/-/-		TBK1_CCD1 domain	–	LP	–
	c.359-1G>A	-/-/-		dbscsnv: 1.000	–	P	–
	T462fs	-/-/-		TBK1_CCD1 domain	–	P	28008748
	T31fs	-/-/-		STKc_TBK1 domain	–	LP	–
	T331N	-/-/-	D/P/D	Ubl_TBK1 domain	–	VUS	–
	M719V	-/-/-	T/B/N	TBK1_CCD1 domain	–	VUS	–
	R271W	-/-/-	D/B/D	STKc_TBK1 domain	–	VUS	–
*GRN*	P439fs	-/-/-			–	LP	–
	P50fs	-/-/-			–	LP	–
	T220I	-/-/-	D/D/D	GRAN domain	–	VUS	–
*VCP*	P188T	-/-/-	D/D/D	CDC48 super family domain	–	LP	–
*TARDBP*	I383V	-/-/0.00005	T/B/N	C-terminal site	LP	LP	18802454
*SQSTM1*	E362K	-/-/-		D/B/D	–	VUS	31859009
*UBQLN2*	P500S	-/-/-		T/B/D	–	VUS	28716533
*DCTN1*	R292H	-/-/-	D/B/D	Smc and Agg_substance	–	VUS	–
*HNRNPA1*	N50S	-/-/-	T/B/D	RRM domain	–	VUS	–

SIFT: D = deleterious, T = tolerated; Polyphen: D = probably damaging, P = possibly damaging, B = benign; Mutationtaster: A = disease_causing_automatic, D = disease_causing, N = polymorphism; Clinvar/ACMG: P = pathogenic, LP = likely pathogenic, VUS = variants of uncertain significance.

### Mutation frequency

**Fig. 1:**
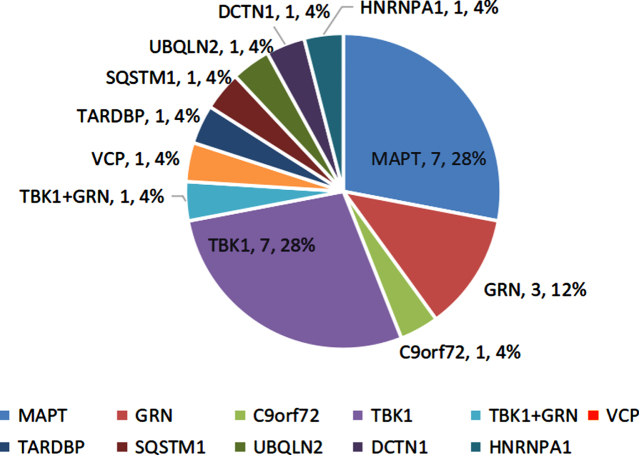
Genetic spectrum of the FTD participants.

11.8% (24/204) participants harbored the potential causative variants in FTD-related genes. Of them, 3.5% (7/204) subjects had the *MAPT* variants, and 3.5% (7/204) had the *TBK1* variants. The remaining 10 cases carried the rare variants in the *GRN* (*n* = 2, 1.0%), *GRN*+*TBK1* (*n* = 1, 0.5%), *VCP* (*n* = 1, 0.5%), *TARDBP* (*n* = 1, 0.5%), *UBQLN2* (*n* = 1, 0.5%), *SQSTM1* (*n* = 1, 0.5%), *DCTN1* (*n* = 1, 0.5%), and *HNRNPA1* (*n* = 1, 0.5%), as well as GGGGCC repeats in the *C9orf72* (*n* = 1, 0.5%) (Fig. 1).

The mutation frequencies of the FTD-genes were higher in the early-onset and familial patients relative to the late-onset and sporadic subjects (14.0%, 16.0% versus 5.6%, 8.9%). The svPPA and nvPPA patients showed higher mutation frequencies than bvFTD patients (22.0%, 19.0% versus 6.1%).

### Phenotype of FTD-gene variant carriers

The clinical characteristics of the 24 FTD-gene variant carriers are shown in Supplementary Table 1. Of them, 54.2% (13/24) subjects had a family history of dementia. 12.5% (3/24) had a family history of MND or schizophrenia.

29.2% (7/24) cases had behavioral/psychiatric symptom as their initial symptom. They were clinically diagnosed with bvFTD. 70.8% (17/24) subjects presented with language impairment in the early disease course. They were diagnosed with nvPPA (*n* = 8) and svPPA (*n* = 9), respectively.

During the disease progression, 88.9% (8/9) svPPA and 62.5% (5/8) nvPPA patients developed behavioral/psychiatric symptom, such as irritability, anxiety, depression, paranoid, apathy, loss of empathy, disinhibition, and stereotyped and compulsive behavior. Conversely, 28.6% (2/7) bvFTD patients showed expressive aphasia. Moreover, 28.6% (2/7) bvFTD, 37.5% (3/8) nvPPA, and 11.1% (1/9) svPPA subjects had pyramidal symptoms. 42.9% (3/7) bvFTD and 11.1% (1/9) svPPA subjects showed extrapyramidal symptoms. One bvFTD and one svPPA patient progressed into MND. 14.3% (1/7) bvFTD, 37.5% (3/8) nvPPA, and 77.8% (7/9) svPPA subjects developed memory deficit. In addition, urinary or fecal symptom was observed in 33.3% (8/24) subjects. Dietary change and sleep disorder were in 16.7% (4/24) and 25.0% (6/24) cases, respectively.

On structural imaging, the temporal and frontal atrophy were the most common, which occurred in 95.8% (23/24) and 62.5% (15/24) subjects, respectively. 16.7% (4/24) cases had parietal atrophy. 54.2% (13/24) subjects showed left hemisphere-predominant atrophy and 25.0% (6/24) showed bilateral symmetrical atrophy, while 20.8% (5/24) exhibited right hemisphere-predominant atrophy or hypometabolism.

### Phenotype of TBK1 variant carriers

**Fig. 2:**
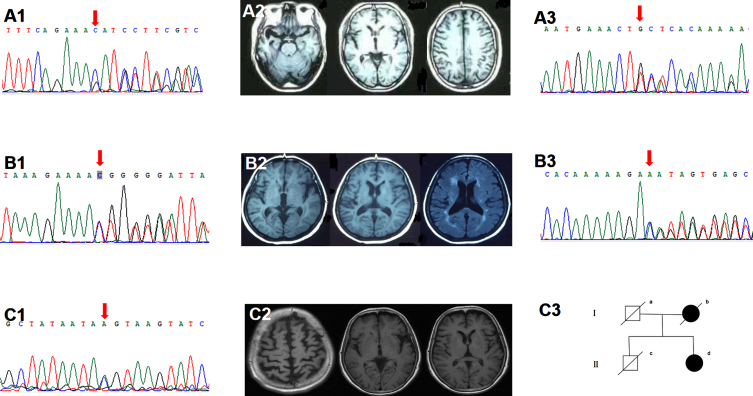
Clinical data of the *TBK1* variant carriers. A1-2) Sanger sequencing indicated the *TBK1* p.K622fs (c.1866_1872del) mutation. Brain MRI showed right temporal predominant atrophy. B1-2) Sanger sequencing indicated the *TBK1* p.T31fs (c.92delC). Brain MRI showed left temporal predominant atrophy with periventricular white matter hyperintensities, fazekas grade 2. A3, B3) Sanger sequencing indicated the *TBK1* p.T457fs (c.1371_1372del), p.T462fs (c.1385_1388del). C1-3) Sanger sequencing indicated the *TBK1* p.T331N (c.992C>A) mutation. Brain MRI showed bilateral frontal and parietal atrophy with mild left insular atrophy. II-d was the index patient. Her mother developed cognitive decline in her 80s and died ten years later. Her father died at 70 without cognitive impairment. Her cognitively healthy brother had a sudden death at 60.

Five subjects harbored the PLPV in the *TBK1*. The *TBK1* T457fs carrier started with stereotyped behavior at 50. In the past ten years, at four o’clock every morning, he got up and bicycled to the same restaurant five kilometers away from home and ordered the same food. Gradually, he developed apathy, loss of empathy, and expressive aphasia. CSF testing showed normal Aβ_42_ (746 pg/ml), p-tau (24pg/ml), and t-tau (150 pg/ml). The *TBK1* T31fs carrier was characterized by an early-onset effortful speech with pyramidal sign. Brain MRI showed left temporal predominant atrophy (Fig. 2). The *TBK1* K622fs carrier started with impaired naming and comprehension at 69. Brain MRI showed right temporal predominant atrophy (Fig. 2). PIB-PET was negative. The *TBK1* c.359-1G>A carrier started with semantic aphasia at 54. His father had cognitive decline after cerebral hemorrhage in his 70 s. The *TBK1* T462fs carrier started with semantic aphasia at 53. Two years later, she developed MND.

Two subjects carried the *TBK1* VUS. The *TBK1* T331N carrier started with an effortful speech at 54. Three years later, she developed personality change, dyscalculia, and gait instability. Physical examination suggested pyramidal involvement. Brain MRI showed bilateral frontal, parietal and left insular atrophy (Fig. 2). CSF testing indicated normal Aβ_42_ (1040 pg/ml) and elevated p-tau (57 pg/ml) and t-tau (243pg/ml). The *TBK1* M719V carrier started with word-finding difficulty and agrammatism at 50. Three years later, she developed loss of empathy and stereotyped behavior. Brain FDG-PET showed left cerebral hypometabolism. Her brother suffered from MND.

### Phenotype of other FTD-gene variant carriers

Seven subjects harbored the *MAPT* L583V, P618L, and Q561P. They were reported by our group before [8]. A 57-year-old female with the *C9orf72* GGGGCC repeats presented with behavioral/psychiatric disturbance. The *UBQLN2* P500S carrier started with apathy, loss of empathy, and dietary change at 57. One year later, she developed MND. Her two sisters suffered from schizophrenia. The *TARDBP* I383V carrier started with semantic aphasia at 53. He became a heavy smoker and drinker. He picked up the cigarette ends on the ground. His mother and brother presented with language impairment and psychotic disorder in their 40 s. The *SQSTM1* E362K carrier started with semantic aphasia at 53. CSF testing indicated normal Aβ_42_ (666 pg/ml), elevated p-tau (58 pg/ml) and t-tau (430 pg/ml).

Six patients carried the novel variants in other FTD genes. The *DCTN1* R292H carrier presented with semantic aphasia at 59. Brain FDG-PET showed left-temporal predominant hypometabolism. CSF testing indicated normal Aβ_42_ (659 pg/ml), p-tau (33 pg/ml), and t-tau (153 pg/ml). The *HNRNPA1* N50S started with loss of empathy, apathy, paranoid, and repetitive behavior at 54. Gradually he developed parkinsonism. FDG-PET showed bilateral frontal hypometabolism. PIB-PET was negative. The *VCP* P188T carrier started with semantic aphasia at 57. Two years later, he developed expressive disorder. He collected rubbish in the public. The *GRN* P50fs carrier presented with non-fluent aphasia, while the *GRN* P439fs carrier exhibited semantic aphasia. The *GRN* T220I carrier also carried the *TBK1* R271W. His mother had cognitive decline in her 70 s. His brother suffered from schizophrenia. He showed loss of empathy, disinhibition, and compulsive behavior at 71.

### Comparisons between MAPT and TBK1 variant carriers

Compared with the *MAPT* patients, the *TBK1* variant carriers showed an older AOO (55.0±6.0 versus 46.1±16.2) and a higher proportion of sporadic cases (71.4% (5/7) versus 14.3% (1/7)). On structural MRI, the *TBK1* carriers showed less frontal but more parietal involvement relative to the *MAPT* carriers (42.9% (3/7) versus 100.0% (7/7); 42.9% (3/7) versus 0.0% (0/7)). Accordingly, 42.9% (3/7) of the *TBK1* variant carriers had parietal damage associated symptom, such as dyscalculia, visuospatial dysfunction.

## DISCUSSION

In this study, the FTD-gene mutations are detected in 11.8% (24/204) FTD patients. These is close to the previous findings. In a Chinese south cohort, 10.9% FTD patients harbored a pathogenic/likely pathogenic variant [16]. Moreover, we find that svPPA and nvPPA patients have higher mutation frequencies than bvFTD patients (22.0%, 19.0% versus 6.1%).

Like the previous genetic studies of Chinese FTD population, the rare variants in the *MAPT*, *GRN*, *TBK1*, *VCP,* and the GGGGCC repeats in the *C9orf72* are found in this study. In addition, the rare variants in the *TARDBP*, *UBQLN2*, *SQSTM1*, *DCTN1*, and *HNRNPA1* are involved in the mutation spectrum.

Seven novel PLPV are found. The *TBK1* T31fs, T457fs, K622fs, c.359-1G>A and the *GRN* P50fs, P439fs are frameshift and splicing mutations. The *VCP* P188T is a missense mutation in the cdc48_2 domain. They are absent in the 1000genome, ESP6500, and GnomAD databases. We reviewed the mutation distribution of the *TBK1*, *GRN*, and *VCP* in the clinvar database (https://www.ncbi.nlm.nih.gov/clinvar). 60.4% (29/48) *TBK1* and 75.8% (75/99) *GRN* PLPV are frameshift, splicing, or stopgain mutations. 57.7% (15/26) *VCP* missense PLPV are located in the cdc48_2 domain. According to the ACMG criteria, the seven variants are novel PLPV.

The *MAPT*, *GRN*, and *C9orf72* account for half of familial FTD cases. Among 58 PPA subjects with autopsies, Mesulam found genetic mutations only in the *GRN*. However, in this study, the *TBK1* shows a high mutation frequency. It is supposed to be one gene frequently harboring FTD-causing variants after the *MAPT*, *GRN*, and *C9orf72* [17–19]. FTD and MND are the most prevalent phenotypes [19]. Herein, the seven *TBK1* variant carriers present with bvFTD or PPA. One case develops MND. Compared with the *MAPT* carriers, the *TBK1* carriers show a later disease onset, as well as a higher incidence of parietal atrophy and parietal damage associatedsymptoms.

The *DCTN1* mutations are mainly linked to Perry syndrome. The *HNRNPA1* mutations are primarily detected in the subjects with inclusion body myopathy, Paget disease. They also account for a small percentage of FTD or MND cases [20, 21]. In this report, the *DCTN1* R292H carrier presents with semantic aphasia and psychiatric symptom. The *HNRNPA1* N50S carrier shows behavioral/psychiatric symptom and parkinsonism. Neither of them developed MND.

The *TARDBP* I383V, *UBQLN2* P500S, and *SQSTM1* E362K were reported in familial MND patients before [12, 14, 15]. In this study, the *UBQLN2* P500S carrier presents with bvFTD and MND. The *TARDBP* I383V and *SQSTM1* E362K are in two svPPA patients. The *MAPT* L583V, P618L, and Q561P were reported by our group. Dr. Mao described the phenotypic heterogeneity of the *MAPT* variant carriers [11]. Taken together, our results suggest the phenotypic heterogeneity of these variants.

Of the 24 FTD-gene variant carriers, 54.2% (13/24) subjects have a family history of dementia. In addition, the *TBK1* M719V carrier has a family history of MND. The *UBQLN2* P500S and the *TBK1* R271W/*GRN* T220I carriers have a family history of schizophrenia. MND and FTD belong to a neurodegenerative disease spectrum. They have some common genetic and pathological bases [22]. FTD and schizophrenia may also have some genetic links in common [23]. The previous studies have confirmed the association between the *GRN* mutations and schizophrenia [24]. The relevance between the *UBQLN2*, *TBK1*, and schizophrenia is unknown.

Compared with nvPPA patients, svPPA cases present with more behavioral/psychiatric and memory symptoms (88.9% (8/9), 77.8% (7/9) versus 62.5% (5/8), 37.5% (3/8)), as well as less pyramidal symptoms (11.1% (1/9) versus 37.5% (3/8)). These are similar to Ulugut’s findings [25] Moreover, we find that bvFTD subjects show more motor symptoms relative to PPA cases (71.4% (5/7) versus 29.4% (5/17)).

Mesulam noted that the most distinctive feature of PPA on structural imaging was left hemisphere-predominant atrophy [26]. However, in this study, the *TBK1* K622fs, the *GRN* P439fs and the *SQSTM1* E362K carriers exhibit right hemisphere-predominant atrophy or hypometabolism. They are all right-handed and present with semantic aphasia, behavioral/psychiatric symptoms, and memory deficit. Ulugut found that 11.3% FTD patients showed right temporal-predominant atrophy [27]. These patients might not be purely TDP type C pathology [28].

In conclusion, this study further confirms the genetic and clinical heterogeneity of Chinese FTD population. The *TBK1* has a high mutation frequency in Chinese FTD patients. The main limitation of this study is the lack of neuropathological confirmation. Functional analysis of the novel rare variants should be performed next. Besides, the subjects in this paper are all Chinese native speakers. However, the PPA diagnostic criteria we used are from English-speaking population. Unlike English, Chinese is a hieroglyphic language. In writing, Chinese PPA patients might show character structural errors instead of letter omissions. Therefore, the diagnostic criteria for Chinese PPA population should be established in thefuture.

## Supplementary Material

Supplementary Material
