# Supplementary material for: Genetic Spectrum and Clinical Heterogeneity of Chinese Frontotemporal Dementia Patients: Data from PUMCH Dementia Cohort
Source: J Alzheimers Dis. 2022 Sep 27;89(3):893–901. doi: 10.3233/JAD-220594 (PMC9535560; doi:10.3233/JAD-220594)
Supplement: Supplementary Material [file jad-89-jad220594-s001.pdf]

Supplementary Material

Genetic Spectrum and Clinical Heterogeneity of Chinese Frontotemporal Dementia Patients: Data from PUMCH Dementia Cohort

Supplementary Table 1. Clinical characteristics of 24 FTD-gene variant carriers

Case	Gene	Variant	Gender	Age /AOO	FHD	Handedness	*APOE*	Clinical manifestation	Clinical diagnosis
1	*MAPT*	L583V	Male	35/31	+	Right	ε3ε4	● An effortful speech with agrammatism, dysgraphia, hypersomnolence, disinhibition, repetitive behavior, memory deficit; MMSE 8, ADL 43;<br>● MRI: left fronto-temporal atrophy; FDG-PET: left fronto-temporal predominant hypometabolism	nvPPA
2	*MAPT*	L583V	Female	32/31	-	Right	ε2ε3	● Personality change, irritability, disinhibition, repetitive and compulsive behavior, pyramidal sign, executive dysfunction; MMSE 16, ADL 32;<br>● MRI: right fronto-temporal predominant atrophy	bvFTD
3	*MAPT*	L583V	Female	34/32	+	Right	ε3ε3	● An effortful speech with agrammatism, pyramidal sign; CDR 2;<br>● MRI: left fronto-temporal atrophy	nvPPA
4	*MAPT*	L583V	Female	41/40	+	Right	ε2ε3	● Impaired naming and comprehension; irritability, depression, insomnia, memory deficit; CDR 3;<br>● MRI: left fronto-temporal atrophy	svPPA
5	*MAPT*	P618L	Male	62/60	+	Right	ε3ε3	● Irritability, restlessness, disinhibition, dietary change, apathy, expressive aphasia, bradykinesia, gait instability, urinary incontinence, executive dysfunction; MMSE 22, ADL 36;<br>● MRI: right fronto-temporal predominant atrophy; CSF: Aβ42 404pg/ml, p-tau 36pg/ml, t-tau 121pg/ml	bvFTD
6	*MAPT*	P618L	Male	63/62	+	Right	ε3ε3	● An effortful speech with agrammatism, apathy, stereotyped behavior; MMSE 8, ADL 45;<br>● MRI: bilateral fronto-temporal atrophy	nvPPA
7	*MAPT*	Q561P	Female	69/67	+	Right	ε3ε3	● An effortful speech with agrammatism; CDR 3;<br>● MRI: bilateral fronto-temporal atrophy with left predominance	nvPPA
8	*TBK1*	T457fs	Male	60/50	-	Right	ε3ε3	● Stereotyped behavior, apathy, loss of empathy, expressive aphasia; MMSE 0, ADL 44;<br>● MRI: bilateral temporal atrophy; CSF: Aβ42 746pg/ml, p-tau 24pg/ml, t-tau 150pg/ml	bvFTD
9	*TBK1*	K622fs	Male	71/69	-	Right	ε3ε3	● Impaired naming and comprehension, memory deficit, paranoid, dietary change; MMSE 23, ADL 26;<br>● MRI: right temporal predominant atrophy; PIB-PET (-)	svPPA

10	*TBK1*	c.359-1G>A	Male	55/54	+	Right	ε3ε3	● Impaired naming and comprehension, apathy, stereotyped behavior, dyscalculia; MMSE 0, ADL 48;<br>● MRI: bilateral fronto-temporo-parietal atrophy with left predominance	svPPA
11	*TBK1*	T462fs	Female	59/53	-	Right	ε3ε4	● Impaired naming and comprehension, MND, memory deficit, constipation, executive dysfunction; MMSE 13, ADL 40;<br>● MRI: bilateral temporal atrophy; FDG-PET: bilateral temporal hypometabolism	svPPA
12	*TBK1*	T31fs	Female	56/55	-	Right	ε3ε4	● An effortful speech, pyramidal sign, memory deficit, urinary incontinence, joint deformity;<br>● Brain: left temporal predominant atrophy, periventricular white matter lesions, Fazekas grade 2	nvPPA
13	*TBK1*	M719V	Female	55/50	-	Right	ε3ε3	● Word-finding difficulty and agrammatism, loss of empathy, stereotyped and compulsive behavior, memory deficit, dyscalculia, urinary incontinence, executive and visuospatial dysfunction; MMSE 21, ADL 29;<br>● MRI: bilateral fronto-temporo-parietal atrophy with left predominance; FDG-PET: left cerebral hypometabolism	nvPPA
14	*TBK1*	T331N	Female	59/54	+	Right	ε2ε3	● An effortful speech with spared comprehension, personality change, dyscalculia, pyramidal sign, sleep-talking, urinary urgency, executive dysfunction; MMSE 25, ADL 38;<br>● MRI: bilateral fronto-parietal atrophy with mild left insular atrophy; CSF: Aβ42 1040pg/ml, p-tau 57pg/ml, t-tau 243pg/ml	nvPPA
15	*TBK1* +*GRN*	R271W +T220I	Male	81/71	+	Right	ε3ε4	● Loss of empathy, disinhibition, compulsive behavior, memory deficit, tremor, hypertonia; MMSE 18, ADL 40;<br>● MRI: bilateral fronto-temporal atrophy	bvFTD
16	*GRN*	P50fs	Male	62/61	-	Right	ε3ε3	● An effortful speech with spared comprehension, personality change, dyscalculia; MMSE 24, ADL 27;<br>● MRI: left fronto-temporo-parietal predominant atrophy	nvPPA
17	*GRN*	P439fs	Female	66/64	-	Right	ε3ε3	● Impaired naming, comprehension and spontaneous speech, dyslexia, dysgraphia, irritability, disinhibition, disorientation, memory deficit, urinary incontinence, visuospatial dysfunction; MMSE 3, ADL 51;<br>● CT: normal; FDG-PET: bilateral parieto-temporal hypometabolism with right predominance; CSF: Aβ42 795pg/ml, p-tau 50pg/ml, t-tau 480pg/ml	svPPA
18	*VCP*	P188T	Male	61/57	-	Right	ε2ε3	● Impaired naming, comprehension and spontaneous speech, inappropriate behavior, memory deficit, disorientation, dyscalculia, tremor; MMSE 13, ADL 29;<br>● MRI: bilateral fronto-temporal atrophy with left predominance	svPPA
19	*TARDBP*	I383V	Male	56/53	+	Right	ε2ε3	● Impaired naming and comprehension, dyslexia, dysgraphia, paranoid, inappropriate behavior, dietary change; CDR 3;<br>● MRI: left fronto-temporal predominant atrophy	svPPA

20	*SQSTM1*	E362K	Male	56/53	-	Right	ε3ε3	● Impaired naming, comprehension and spontaneous speech, dysgraphia, depression, stereotyped behavior, hallucination, memory deficit, insomnia; MMSE 2, ADL 59;<br>● MRI: bilateral temporal atrophy with right predominance; CSF: Aβ42 666pg/ml, p-tau 58pg/ml, t-tau 430pg/ml	svPPA
21	*UBQLN2*	P500S	Female	59/57	-	Right	ε3ε3	● Apathy, loss of empathy, inappropriate behavior, dietary change, constipation, MND; CDR 3;<br>● MRI: left temporal atrophy	bvFTD
22	*DCTN1*	R292H	Male	61/59	-	Right	ε3ε3	● Impaired naming and comprehension, dyslexia, dysgraphia, irritability, anxiety, insomnia, memory deficit; MMSE 21, ADL 31;<br>● MRI: left temporal predominant atrophy; FDG-PET: left temporal predominant hypometabolism; CSF: Aβ42 659pg/ml, p-tau 33pg/ml, t-tau 153pg/ml	svPPA
23	*HNRNPA1*	N50S	Male	56/54	-	Right	ε3ε3	● Apathy, loss of empathy, paranoid, repetitive behavior, bradykinesia, hypertonia, hypersomnia, rapid eye movement sleep behavior disorder, fecal incontinence, increased eating, executive dysfunction; MMSE 23, ADL 27;<br>● MRI: bilateral insular atrophy; FDG-PET: bilateral frontal hypometabolism; PIB-PET (-); CSF: Aβ42 373pg/ml, p-tau 22pg/ml, t-tau 99pg/ml	bvFTD
24	*C9orf72*	GGGGCC repeats	Female	57/54	+	Right	ε3ε3	● Personality and mood change, behavioral disorder; MMSE 27, ADL 24;<br>● MRI: bilateral front-temporal atrophy	bvFTD

bvFTD, behavioral variant of FTD; nvPPA, nonfluent/agrammatic variant of primary progressive aphasia; svPPA, semantic variant of primary progressive aphasia; AOO, age of onset; FHD, family history of dementia; *APOE*, apolipoprotein E; CDR, clinical dementia rating; MMSE, Mini-Mental State Examination; ADL, Activities of Daily Living; CSF, cerebrospinal fluid.
